# Traumatic Distress of COVID-19 and Depression in the General Population: Exploring the Role of Resilience, Anxiety, and Hope

**DOI:** 10.3390/ijerph18168485

**Published:** 2021-08-11

**Authors:** Finiki Nearchou, Ellen Douglas

**Affiliations:** School of Psychology, University College Dublin, 4 Dublin, Ireland; ellen.douglas@ucdconnect.ie

**Keywords:** impact, pandemic, COVID-19, psychological, resilience, mediation, coronavirus, trauma

## Abstract

International evidence published so far shows that the COVID-19 pandemic has negatively impacted on global mental health. Specifically, there is some research suggesting that the psychological distress related to depression, anxiety and posttraumatic stress has impacted on the psychological well-being of the general population. Yet, there is limited evidence on the relational paths between COVID-19 traumatic distress and depression. Participants of this cross-sectional study were 456 adults 18 years old or older from the general population (Mean age = 41.2 years, SD = 11.7) who completed an online questionnaire including measures assessing depression, anxiety, resilience, hope and traumatic distress related to COVID-19. Structural equation modelling was applied to examine the proposed mediation model. The results confirmed the proposed model, with traumatic distress of COVID-19, resilience, anxiety and hope explaining a considerable amount of variance (59%) in depression scores. Traumatic distress of COVID-19 was a strong positive predictor of depression, while anxiety, hope and resilience were both joint and unique mediators of this relationship. Exposure to the COVID-19 pandemic is strongly associated with depression in adults of the general population. The co-occurrence of anxiety may negatively contribute to experiencing higher levels of depression, while resilience and hope may act as buffers against depression associated with the impact of this pandemic. Our findings suggest that wide community-based interventions designed to promote resilience, build hope and reduce anxiety may help mitigate depression associated with exposure to the COVID-19 pandemic.

## 1. Introduction

On 30 January 2020, the World Health Organisation declared the novel coronavirus outbreak a public health emergency of international concern and within few weeks, on 11 March 2020, declared the coronavirus disease 2019 (COVID-19) a global pandemic [1]. This global public health crisis carried detrimental repercussions for societies and economies, affecting every aspect of human life [2]. Health systems were subjected under extreme strain [3,4] and many governments, in an effort to control spread and flatten the curve, imposed severe restrictions including long-term lockdowns, travel restrictions, ban of social gatherings, school closures and mandatory quarantines. Self-isolation and social distancing became the new everyday reality for many citizens around the world, which has been associated with loneliness and psychological distress [5].

International evidence published so far shows that the COVID-19 pandemic has negatively impacted on global mental health [5,6,7,8], confirming concerns expressed very early in the outbreak about subsequent mental health consequences arising from this imminent global health crisis [9]. Although a significant amount of research examining the psychological impact of the pandemic has focused on healthcare workers [10,11,12] and clinical populations [13,14,15,16,17,18], there is some evidence suggesting that the psychological distress related to depression, anxiety and posttraumatic stress has impacted the general population as well. A recent meta-analytic review of 23 studies in the general population found that nearly half of participants surveyed in those studies experienced significant psychological impact during the pandemic, with reported prevalence rates reaching 15%, 24% and 26% for posttraumatic stress symptoms (PTSS), depression and anxiety levels, respectively [6]. Another meta-analysis found a prevalence estimate of 31.9% for anxiety in 17 studies and of 33.7% for depression in 14 studies conducted in the general population during the pandemic [7]. Age and gender have been frequently reported as factors associated with mental health problems during the COVID-19 pandemic. Evidence shows that younger adults (< 40 years old) are generally at higher risk of experiencing higher levels of depression and anxiety amidst the pandemic [7,8,19,20], while older adults may be more susceptible in experiencing posttraumatic stress symptoms [21]. Being a woman has also been reported as a risk factor of experiencing higher depression, anxiety and posttraumatic stress symptoms during this pandemic [8,19,20,22].

Prevalence estimates of depression, anxiety and traumatic distress symptoms during the pandemic tend to vary across studies, mainly because of the different psychometric tools used to measure these constructs. For example, a systematic review found that reported depression rates ranged from 14.6% to 48.3%, anxiety rates ranged from 6.33% to 50%, and traumatic distress symptoms ranged from 7% to 53.8% in studies conducted in the general population during the pandemic [8]. Despite the varying reported rates of these symptoms, findings from these systematic reviews suggest that COVID-19 has impacted on the psychological well-being of the general public. It has also been suggested that in the aftermath of this pandemic, clinicians should be prepared for dealing with the possibility of emerging depressive, anxiety and posttraumatic stress disorders [23]. This indicates that there is an imperative need to obtain more empirical evidence on depression, anxiety and traumatic distress symptoms during the pandemic that can help improve our knowledge on this area.

### 1.1. The Mediating Role of Resilience and Hope

Resilience is the dynamic process of adaptation and coping in the light of exposure to adversities, including diverse stressors from childhood maltreatment and living in extreme poverty to natural disasters and epidemics. Drawing from a socio-ecological framework, resilience lies in the capacity of the individuals to navigate themselves to resources that can support their well-being, located in their surrounding physical and social ecologies (systems). In this framework of a socio-ecological conceptualisation, resilience also lies in the capacity of systems such as families, communities and governments, to provide these resources in contextually and culturally meaningful ways [24,25]. Hence, rather than a personal quality of the individual, resilience is better understood as the process embedded in the dynamic interaction between the individual and the surrounding systems [26].

Over the past decades, a cumulative body of evidence indicated that despite the mental distress following exposure to different types of stressors or traumatic events, the presence of specific factors associated with resilience acted as protective agents and reduced the negative impact of the stressor on the individuals’ psychological well-being. For example, survivors of natural disasters and/or major public events who reported higher levels of resilience were more likely to report lower levels of depression and posttraumatic stress disorder symptoms [27,28,29]. In the light of the COVID-19 pandemic, emerging evidence, though limited, indicates that resilience is negatively associated with depression in adults from the general population [30,31,32,33,34,35]. While this potentially suggests that higher levels of resilience can be associated with lower levels of depression during this pandemic [36], the limited existing evidence does not allow further inferences about the protective role of resilience against depression to be made. Hence, we need to better understand the paths between traumatic distress of COVID-19 and depression by investigating the potential mediating role of resilience.

Snyder operationalised hope as a cognitive goal-oriented process relying on the perception that goals can be met [37]. Research shows that hope can contribute to improved physical and mental well-being [38] and facilitate adjustment when dealing with stressors e.g., [39]. Hope has been identified as a protective factor against depression symptoms associated with negative life events [40,41], natural disasters [42], psychiatric disorders [43,44] and chronic illness [45]. In the context of the COVID-19 pandemic, there is some evidence indicating that higher levels of hope are associated with lower levels of anxiety [46] and COVID-19 stress [47], while a study conducted across 11 countries found that hope was negatively associated with depression [48]. These findings suggest that hope may play a role in reducing psychological distress including depression experienced during this pandemic. However, there is very limited research exploring the relationship between hope and depression during the ongoing COVID-19 pandemic. Hope has also been well established as a factor associated with the process of resilience following exposure to adversities [49,50,51,52]. Notably, prior research showed that hope can be increased through different therapeutic approaches or interventions [53,54,55]. Thus, shedding light into the role of hope as a potential factor that promotes resilience, can add to our knowledge on how this construct may be considered to inform the design of interventions targeting depression during this pandemic.

Research shows that exposure to stressful events can evoke anxiety, which in turn can operate as a contributing factor to experiencing depression. Indeed, some empirical evidence, although limited, suggests that the pathway between exposure to stress and depression can be better understood through the emotional and cognitive functions related to anxiety [56,57,58]. For example, Anyan et al. [59] showed that anxiety mediates the relationship between exposure to stressful events and depression through the formation of negative anxiety-related cognitions and emotions, which in turn can contribute to an increased risk of depression. The mediating role of anxiety in the link between stress and depression was also corroborated by a recent study conducted during the COVID-19 pandemic, showing that anxiety partially mediated the effect of stress on depression [60]. Hence, obtaining a deeper insight into the mediating role of anxiety can help improve our knowledge on further understanding the pathways between COVID-19-related traumatic distress and depression, as well as expand the literature on the mediating role of anxiety.

### 1.2. The Present Study

**Figure 1:**
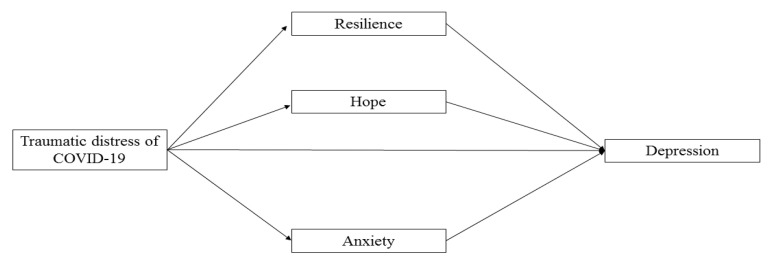
Representation of the hypothesized mediation model. Traumatic distress of COVID-19 is expected to influence depression levels, while resilience, hope and anxiety are expected to mediate this influence.

There is some evidence suggesting an increase in depressive symptoms in the general population during the COVID-19 pandemic in comparison to the pre-pandemic era [8,33,36]. Exposure to traumatic events has been previously linked to increased likelihood of presenting with comorbid anxiety and depressive disorders [61,62]. Thus, informing prevention and intervention policy decisions to tackle depression necessitates the need to gain a better insight into the relationship between the traumatic distress related to COVID-19 and depression. Prior research suggests that resilience following exposure to adversities may enable individuals to cope and continue functioning within normal boundaries. Drawing from a socio-ecological framework, the present study aimed to investigate the mediating role of resilience in the impact of COVID-19 on depression by testing the hypothesized model represented in Figure 1. Because hope has been suggested as a protective factor against depression contributing to resilience following exposure to traumatic events [63], it was included in the model as a potential mediator. Finally, because there is some evidence, although limited, on the mediating role of anxiety in the relationship between traumatic distress and depression, we also included anxiety as one of the mediating variables in this model. Hence, this could enable better understanding of the impact of COVID-19 on depression.

## 2. Methods

### 2.1. Participants

Participants of this cross-sectional study were adults (*n* = 456) from the general population recruited from the island of Ireland (Republic of Ireland and Northern Ireland) and the United States (U.S.). Participants’ age ranged from 18 to 71 years old (M = 41.2 years, SD = 11.7), 62.3% were females (*n* = 284) and 56.8% were from the U.S (*n* = 259). Most participants were married/in a relationship (56%, *n* = 255) and living with family/partner (*n* = 380, 82.3%). Table 1 presents the detailed demographic profile of the study sample.

### 2.2. Measures

#### 2.2.1. Depression

Depression was measured using the depression subscale of the Depresion, Anxiety and Stress Scale–21 (DASS-21) [64]. The DASS-21 is the short version of the original self-report scale (DASS-42), and assesses emotional states related to depression, anxiety and stress. The depression subcale of the DASS-21 includes seven items scored on a four-point Likert scale ranging from 0 (‘Never’) to 3 (‘Almost always’), and asks participants to rate how much each item applied to them during the past week. An example item is ‘I was unable to become enthusiastic about anything’. The total score of the depression subcale is derived by adding the seven items and then multiplying their sum by two. The total score ranges from 0 to 42, with higher scores indicating higher levels of depression. The Cronbach’s alpha indicated an excellent reliability for the present sample (*α* = 0.91).

#### 2.2.2. Anxiety

Anxiety was measured using the anxiety subcale of the DASS-21 [64]. The anxiety subcale of the DASS-21 includes seven items scored on a four-point Likert scale ranging from 0 (‘Never’) to 3 (‘Almost always’) and asks participants to rate how much each item applied to them during the past week. An example item is ‘I was aware of dryness of my mouth’. The total score of the anxiety subcale is derived by adding the seven items and then multiplying their sum by two. The total score ranges from 0 to 42, with higher scores indicating higher levels of anxiety. The Cronbach’s alpha indicated an excellent reliability for the present sample (*α* = 0.91).

#### 2.2.3. Resilience

Resilience was measured using the self-report scale Adult Resilience Measure-Revised (ARM-R) [65]. The ARM-R includes 17 items asking participants to rate the extent to which each statement measuring socio-ecological resilience applies to them. The items fall under two subscales reflecting relational resilience and personal resilience. Example items are ‘I co-operate with people around me’ (personal resilience) and ‘I talk to my family/partner about how I feel’ (relational resilience). For the present study we used the five-point simplified version of the tool [65], with each item scored on a Likert scale ranging from 1 (‘Not at all’) to 5 (‘A lot’). The scale yields a total sum score of resilience ranging from 17–85, with higher scores indicating higher levels of resilience, as well as a score for each subscale. For the purposes of the present study, only the total score of the scale was used in the analysis. The Cronbach’s alpha indicated an excellent reliability for the present sample (*α* = 0.91).

#### 2.2.4. Hope

Hope was measured using the Adult Hope Scale (AHS), a self-report measure developed by Snyder and colleagues [66] to assess the cognitive model of hope. The scale includes 12 items, with four items reflecting pathways and four items reflecting agency, while four items are used as filler items. Participants are asked to rate how well each item describes them on an 8-point Likert scale ranging from 1 (‘Definitely false) to 8 (‘Definitely true). Example items include ‘My past experiences prepared me well for my future’ (agency) and ‘There are lots of ways around any problem’ (pathways). The AHS can be either used to measure levels of the construct of hope or to yield two additional scores from the two subscales measuring agency and pathways. The two subscales reflect the latent factor of hope [67]. For the purposes of the present study, we only measured the latent construct of hope. A total score of hope is calculated by adding the 8 items of the two subscales without including the filler items. The total score ranges from 8 to 64, with higher scores indicating higher levels of hope. The Cronbach’s alpha indicated an excellent reliability for the present sample (*α* = 0.89).

#### 2.2.5. Traumatic Distress Related to COVID-19

The traumatic distress of COVID-19 was measured using the Impact of Event Scale- Revised (IES-R) [68]. The IES-R includes 22 items falling under three subscales and reflecting hyperarousal (seven items), intrusion (seven items) and avoidance (eight items). Although not a diagnostic tool, the IES-R was developed and validated using a specific traumatic event as a reference in the introduction to the individual within a specific time frame of the past seven days. For the purposes of the present study, we indicated the COVID-19 pandemic as the traumatic event in reference. The IES-R has been widely employed to measure posttraumatic stress related to COVID-19, including both general and clinical populations [69,70,71]. Items are rated on a five-point Likert scale ranging from 0 (‘Not at all’) to 4 (‘Extremely’). An example item is ‘I tried not to talk about it’. The scale yields a total score ranging from 0 to 88, with higher scores indicating higher levels of traumatic distress related to COVID-19. The Cronbach’s alpha indicated an excellent reliability for the present sample (*α* = 0.95)

### 2.3. Procedure

Data were collected online via the Qualtrics survey software between 4 August 2020 and 2 October 2020, mainly using purposive sampling. The survey was advertised through different networks and through social media. Snowball sampling was also used, especially through social media. Participants who were 18 years old or older were eligible to participate once they had provided electronic consent. The study was granted ethical approval from the University College Dublin Human Research Ethics Committee-Humanities (HS-20-47-Douglas-Nearchou).

### 2.4. Data Analysis Overview

Descriptive statistics and Pearson product moment correlations were performed for the study variables using the IBM SPSS Statistics for Windows, Version 26 (IBM Corporation Armonk, NY, USA). Structural equation modelling (SEM) was applied to examine whether the theoretical model fitted the data of the present study using the IBM AMOS Version 24(IBM Corporation Armonk, NY, USA). A number of indices and criteria were used to examine the model fitting adequacy. A non-significant chi; the comparative fit index (CFI) and Tucker–Lewis index (TLI), with values > 0.90 indicating a good fit and with values around 0.95 indicating an excellent fit; the root mean square error of approximation (RMSEA) and the standardized root mean square residual (SRMR) with values < 0.08 indicate a good model fit, while values < 0.05 indicate an excellent model fit [72]. Maximum likelihood estimation was used with bias-corrected bootstrapping (*n* = 5000) and 95% confidence intervals (CI). Multigroup analysis was then applied to test whether the model differed between Irish and U.S. participants. A complete dataset was used.

## 3. Results

### 3.1. Descriptive Statistics and Intercorrelations

Table 2 presents levels of anxiety and depression measured by the DASS-21, and levels of traumatic distress related to COVID-19 measured by the IES-R. These levels were calculated according to the cut-off scores of the respective tools. For traumatic distress measured by IES-R, 75.9% of participants reported normal levels, while 74.6% and 62.9% reported normal levels on the DASS-21 scales assessing anxiety and depression levels, respectively. In relation to severity of depression levels, 4.6% reported severe and extremely severe levels, while in relation to anxiety, 4.4% and 4.2% reported severe and extremely severe levels, respectively. A total 7.7% of participants reported experiencing severe levels of traumatic distress. We applied a series of one-way analysis of variance (ANOVA) to examine gender differences and differences by country of residence across all study variables (see Supplementary Materials Table S1 for a detailed presentation of these findings). Despite obtaining a *p*-value < 0.05 for some measures, the very small effect sizes indicated that there were no substantial gender differences in our sample. However, we found differences between Irish and U.S. participants across all variables, with U.S. participants reporting higher mean scores of resilience and hope, and lower mean scores of depression, anxiety and COVID-19 traumatic distress than their counterparts. Table 3 presents means, standard deviations and the correlation matrix of all study variables with significant correlations being evident among all variables.

### 3.2. The Mediating Role of Anxiety, Hope and Resilience

The SEM results indicated that the proposed model has an excellent fit to the data. Chi-square was not significant, *χ^2^* (2) = 4.79, *p* = 0.091, and all other indices further confirmed the excellent fit of the structural model: CFI = 0.99, TLI = 0.98, RMSEA = 0.05, SRMR = 0.024. In total, the model explained 59% of the variance in depression scores (*p* = 0.001, CI = 0.53 to 0.64). The COVID-19 traumatic distress explained 43% of the variance in anxiety scores (*p* = 0.001, CI = 0.36 to 0.50), 10% of the variance in hope scores (*p* < 0.001, CI = 0.06 to 0.15) and 13% of the variance in resilience scores (*p* < 0.001, CI = 0.08 to 0.18).

**Figure 2:**
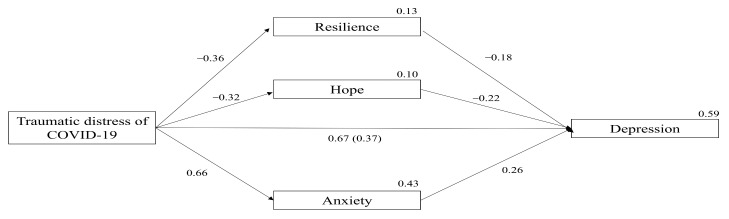
Mediation analyses predicting depression. Values on paths represent standardized β coefficients. Values on endogenous variables represent variance. Value in parenthesis represents the direct effect after considering all mediators in the model. All values are significant.

Figure 2 illustrates the path coefficients and the variances of the proposed model. The COVID-19 traumatic distress significantly predicted all the mediating variables. Specifically, the COVID−19 traumatic distress was a positive strong predictor of anxiety (*β* = 0.66, SE = 0.03, *p* = 0.001, CI = 0.58 to 0.72), and a negative predictor of hope (*β* = −0.32, SE = 0.05, *p* < 0.001, CI = −0.40 to −0.22) and resilience (*β* = −0.36, SE = 0.04 *p* < 0.001, CI = −0.45 to −0.27). In turn all mediators significantly predicted depression scores. Specifically, anxiety was a positive predictor of depression (*β* = 0.26, SE = 0.05, *p* < 0.001, CI = 0.15 to 0.36), while hope (*β* = −0.22, SE = 0.04, *p* < 0.001, CI = −0.30 to −0.14) and resilience (*β* = −0.18, SE = 0.04, *p* < 0.001, CI = −0.27 to −0.09) were negatively associated with depression.

The COVID-19 traumatic distress had a total strong significant effect on depression prior to considering any mediating effects by positively predicting depression scores (*β* = 0.67, SE = 0.03, *p* = 0.001, CI = 0.61 to 0.72). The bootstrapped mediation analysis showed that when all mediators were considered in the model, the effect of the traumatic distress of COVID-19 was reduced but still exerted a significant positive effect on depression (*β* = 0.37, SE = 0.05, *p* < 0.001, CI = 0.27 to 0.47). Further inspection of the indirect effect revealed that this was significant (*β* = 0.30, SE = 0.04, *p* < 0.001, CI = 0.22 to 0.38), indicating that when considered jointly, resilience, hope and anxiety mediated the effect of the COVID-19 traumatic distress on depression. Because this effect remained significant after considering the mediators, a partial mediation was present.

The next step was to examine the unique indirect effects of each of the three mediators of the model. Anxiety was the stronger mediator of the effect of COVID-19 traumatic distress on depression (*β* = 0.10, SE = 0.02, *p* < 0.001, CI = 0.06 to 0.14), while hope (*β* = 0.04, SE = 0.01, *p* < 0.001, CI = 0.02 to 0.07) and resilience (*β* = 0.039, SE = 0.01, *p* < 0.001, CI = 0.02 to 0.06) showed almost equal mediating effects. The final step was to examine whether the SEM model fit equally to data from Irish and U.S. participants. Multigroup analysis showed that there were no differences in the structural weights of the model between the two groups of participants Δ*χ*^2^ (1) = 0.09, *p* = 0.76.

## 4. Discussion

This study aimed to explore the relationship between the COVID-19 traumatic distress and depression in the general population. It also aimed to further our understanding of this relationship by considering resilience, hope and anxiety as mediating variables. The SEM results confirmed the proposed model with traumatic distress of COVID-19, resilience, anxiety and hope explaining a considerable amount of variance (59%) in depression scores. Specifically, our findings showed that the traumatic distress of COVID-19 measured by the IES-R had a strong positive effect on depression, which was mediated by resilience, anxiety and hope. Notably, anxiety was the strongest unique mediator of this relationship, followed equally by resilience and hope. This suggests that exposure to the COVID-19 pandemic is strongly associated with depression in adults of the general population. It also suggests that the co-occurrence of anxiety may negatively contribute to experiencing higher levels of depression, while resilience and hope may act as buffers against depression associated with the impact of this pandemic. Finally, the multigroup analysis showed no differences between Irish and U.S. participants, which suggests that the same patterns of relationships between the study variables apply to both groups.

The COVID-19 traumatic distress was a strong predictor of depression and anxiety in our sample, which adds to existing evidence indicating that exposure to this pandemic is positively linked to depression and anxiety in the general public [73]. Notably, available evidence on mental health consequences of the pandemic on general population derives from studies conducted in heterogenous cultural and ethnic contexts and at different chronological points over the course of the pandemic. Our findings add to this pool of knowledge, which further confirms the pervasiveness and the seriousness of the repercussions of the pandemic on global public mental health. Considering that depression has been identified as a major public health concern by pre-pandemic research [74,75], our findings also highlight the need to urgently prioritize actions to tackle depression linked to the COVID-19 outbreak.

Anxiety was the strongest unique mediator of the relationship between COVID-19-related traumatic distress and depression. This adds to the existing limited pool of evidence showing the mediating role of anxiety in the relationship between traumatic distress and depression, e.g., [59,60]. The comorbidity and the bilateral association of anxiety and depression has been well established in the pre-pandemic literature [76,77,78], which has been further corroborated by studies that assessed depression and anxiety levels during this pandemic [73]. However, our finding showing that anxiety mediates the relationship between the traumatic distress of COVID-19 and depression sheds light on the way that anxiety may operate in the light of exposure to this specific traumatic event. Because emerging evidence shows that anxiety related to the COVID-19 pandemic affects the general population [79], this finding suggests that there is an increased likelihood for those experiencing anxiety to also experience increased levels of depression during this pandemic. Hence, aiming to reduce anxiety through targeted interventions developed for the general population may contribute to the prevention or decrease the likelihood of experiencing increased depression levels during the COVID-19 pandemic.

Most participants reported experiencing normal levels of depression (62.9%) and anxiety (74.6%). A little less than one third of participants reported experiencing mild/moderate levels of depression (27.9%), while just under one in five participants reported experiencing mild/moderate levels of anxiety (16.6%). The percentage of participants who reported experiencing severe/extremely severe levels was quite similar for depression (9.2%) and anxiety (8.6%). These findings are broadly consistent with findings reported by other studies that measured depression and anxiety using the respective DASS-21 subscales [69,80]. Our finding classifying most participants’ responses within the normal range of depression and anxiety levels is consistent with other studies that measured depression and anxiety levels during the pandemic using different psychometric tools [23]. In terms of COVID-19 traumatic distress levels, 76% of our participants reported experiencing normal levels, which is consistent with some studies that used the IES-R [69,81], but not consistent with other studies that employed the same tool [80,82]. These discrepancies in findings across studies suggest that it is imperative to measure levels of COVID-19 traumatic distress frequently, across different time points and across different contexts.

Because the pandemic has evolved into a long-term global health emergency with restrictions and disruptions in daily life remaining in force, the mental health consequences of this are likely to be persisting and to continue affecting large cohorts of the global population. This realisation becomes increasingly important when considered within the context of COVID-19 health anxiety in relation to which serious concerns have been raised. These concerns refer to the extent to which this specific type of health anxiety could continue affecting individuals in the long-term aftermath of this pandemic [83]. COVID-19 health anxiety differs from ordinary health anxiety because, in essence, the coronavirus disease (COVID-19) has unique characteristics when compared to other epidemics or health phenomena, such as extended media coverage, higher transmission and lethality rates and symptoms resembling those of anxiety (e.g., shortness of breath and persistent chest pressure and/or pain) [83,84]. Indeed, it has been suggested that the psychological impact of COVID-19 might be more severe for those already experiencing health anxiety [84]. Our findings showed that anxiety was the strongest unique mediator in explaining the relationship between traumatic distress and depression. Taken together, these may suggest that the role of anxiety in explaining this relationship may even be more significant and more complicated as different anxiety constructs may be intertwined. Future research should address this by assessing and comparing the role of different constructs of anxiety (e.g., health anxiety and general anxiety) in relation to this pandemic.

Resilience and hope were also each a unique mediator in our model, which suggests that despite the distress associated with exposure to this pandemic, to sustain their well-being individuals navigated themselves to resources that were available to them. Through the socio-ecological lens, resilience is promoted via the interaction between the individual and the system(s) within which resources of support can be made available. Enhancing these resources and/or maximizing access can facilitate individuals to interact and use those to sustain their well-being. This can be considered in the light of clinical implications for mental health care professionals and especially for psychiatrists who are at the forefront of the mental health response to this pandemic [85]. Psychological treatments such as acceptance and commitment therapy can be valuable tools in the psychiatrists’ arsenal, as it has been proved quite effective in treating depression and anxiety [86], especially when pharmacological treatment is not deemed appropriate [83]. Within this context, our findings can also be used to inform the design of wide community-based interventions designed to promote resilience, build hope and reduce anxiety, which may help mitigate depression associated with exposure to the COVID-19 pandemic. For example, online [87,88] or in-person [89,90,91] mindfulness-based interventions may offer a flexible as well as an effective approach to reduce anxiety and depression levels, while other forms of online supports such as virtual support groups [92] and self-administered web-based interventions [93] may also facilitate wider access and promote psychological well-being.

Another implication concerns the promotion of community-based resilience indirectly, through enhancing the provision of mental health services and responding to the needs of individual mental health providers emerged from this pandemic. For instance, psychiatrists, through their role, are well-placed to guide adaptations in response to this evolving crisis, as well as lead on the delivery of public mental health approaches [85,94] that can promote community-wide resilience and act protectively against the impact of COVID-19 distress.

Despite its strengths, the present study has some limitations that should be considered when interpreting the results. First, we applied a cross-sectional design which does not allow to capture potential changes in the levels of hope, resilience, depression, anxiety and COVID-19 traumatic distress that could correspond to different time points of the pandemic. For example, studies that measured traumatic distress related to COVID-19 during the first months since the outbreak reported higher levels than those reported by our study, e.g., [80]. Future studies should consider applying longitudinal designs to monitor changes on these variables over time. Second, because we measured depression and anxiety only at one time point after the COVID-19 outbreak, any inferences regarding changes on these variables before and after the pandemic should be avoided. Third, while we measured resilience through a socio-ecological perspective, we did not explicitly measure potential sources of supports related to this pandemic that could be located across different systems. For example, several easily accessible online mental health resources have emerged during this pandemic, while the online provision of mental health services due to COVID-19 restrictions has become very common. This may have facilitated individuals to access these resources, which in turn may have contributed to improve or sustain their well-being. Future studies could address this by measuring formal and informal help-seeking intentions and behaviours during the pandemic, as well as by examining the role of virtual mental health resources and supports in promoting resilience. Finally, because our sample size was moderate, we warrant caution when interpreting and considering generalisation of our findings. Future research could provide additional support to the present study.

## 5. Conclusions

The present study showed that COVID-19 traumatic distress was a strong predictor of depression in the general population. Anxiety was the strongest unique mediator of this relationship, suggesting that experiencing higher levels of anxiety may contribute to experiencing higher levels of depression related to the COVID-19 traumatic distress. Resilience and hope were also unique mediators of this relationship, thus may act as protective factors against the negative impact of COVID-19 traumatic distress on depression. Our findings highlight that exposure to this pandemic has negative psychological impact on the general population. They also highlight that this impact may be counteracted by delivering wide and easily accessible community-based interventions aiming to promote resilience, build hope and reduce anxiety.

## Figures and Tables

**Table 1:** Demographic profile of the study sample (*n* = 456).

	*n*	%
Gender		
Males	171	37.5%
Females	284	62.3%
Not identify as female/male	1	0.2%
Country of residence		
Republic of Ireland/Northern Ireland	197	43.2%
United States	259	56.8%
Marital status		
Single	165	36.2%
Married/relationship	255	56%
Separated/divorced/widowed	36	7.8%
Living situation		
Living on their own	55	12.1%
Living with others (not family)	21	4.6%
Living with family/partner	380	83.2%

**Table 2:** Levels of anxiety, depression and traumatic distress related to COVID-19 in the study sample (*n* = 456).

	Normal (*n*) %	Mild (*n*) %	Moderate (*n*) %	Severe (*n*) %	Extremely Severe (*n*) %
Traumatic distress	(346) 75.9%	(56) 12.3%	(19) 4.1%	(35) 7.7%	
Anxiety	(340) 74.6%	(27) 5.9%	(50) 11%	(20) 4.4%	(19) 4.2%
Depression	(287) 62.9%	(56) 12.3%	(71) 15.6%	(21) 4.6%	(21) 4.6%

Traumatic distress measured by the Impact of Event Scale-Revised, anxiety and depression measured by the respective subscales of Depression Anxiety and Stress Scale–21. Anxiety cut off scores: normal (0–7), mild (8–9), moderate (10–14), severe (15–19), extremely severe (≥20). Depression cut off scores: normal (0–9), mild (10–13), moderate (14–20), severe (21–27), extremely severe (≥28). Traumatic distress: normal (0–23), mild (24–32), moderate (33–38), severe (≥39).

**Table 3:** Correlation matrix, means and standard deviations of all study variables (*n* = 456).

Variables	M	SD	1	2	3	4
1. Traumatic distress of COVID-19	14.8	14.5	_			
2. Resilience	71.5	10.1	−0.36 *	_		
3. Anxiety	4.89	6.44	0.66 *	−0.29 *	_	
4. Hope	51.1	8.61	−0.32 *	0.59 *	−0.28 *	_
5. Depression	8.33	8.71	0.67 *	−0.51 *	0.61 *	−0.51 *

M, mean scores; SD, standard deviation. * *p* < 0.001.

## Data Availability

No data are publicly available for this study due to ethical restrictions.

## Supplementary Materials

The following are available online at https://www.mdpi.com/article/10.3390/ijerph18168485/s1, Table S1: Mean scores, standard deviations and one way analysis of variance results accounted for by gender and country of residence.
