# Supplementary material for: Traumatic Distress of COVID-19 and Depression in the General Population: Exploring the Role of Resilience, Anxiety, and Hope
Source: Int J Environ Res Public Health. 2021 Aug 11;18(16):8485. doi: 10.3390/ijerph18168485 (PMC8394400; doi:10.3390/ijerph18168485)
Supplement: Supplementary file 1 [file ijerph-18-08485-s001.zip › ijerph-1300177-supplementary.pdf]

**Table S1.** Mean scores, standard deviations and one way analysis of variance results accounted for by gender and country of residence.

| **Gender** | **Traumatic distress (M, SD)** | **Anxiety (M, SD)** | **Depression (M, SD)** | **Resilience (M, SD)** | **Hope (M, SD)** |
|---|---|---|---|---|---|
| Female | 16.1 (14.8) | 5.57 (7.04) | 8.99 (9.10) | 71.8 (10.0) | 50.6 (9.02) |
| Male | 12.7 (14.1) | 3.67 (5.02) | 7.20 (7.80) | 70.1 (10.0) | 52.1 (7.69) |
| ANOVA ($n$= 455) | $F$ (1, 454) = 6.06, $p$ = 0.014, $\eta^2$ = 0.01 | $F$ (1, 454) = 9.50, $p$ = 0.002, $\eta^2$ = 0.02 | $F$ (1, 454) = 4.53, $p$ = 0.03, $\eta^2$ = 0.009 | $F$ (1, 455) = 0.76, $p$ = 0.38, $\eta^2$ = 0.001 | $F$ (1, 455) = 3.15, $p$ = 0.08, $\eta^2$ = 0.006 |
| Country of residence | | | | | |
| Ireland | 18.7 (16.2) | 6.24 (7.3) | 11.1 (9.59) | 69.2 (10.6) | 48.4 (9.12) |
| U.S. | 11.9 (12.4) | 3.85 (5.45) | 6.19 (7.29) | 73.1 (9.27) | 53.1 (7.62) |
| ANOVA ($n$ = 456) | $F$ (1, 455) = 26.3, $p$ < 0.001 $\eta^2$ = 0.05 | $F$ (1, 455) = 15.9, $p$ < 0.001 $\eta^2$ = 0.03 | $F$ (1, 455) = 39.1, $p$ < 0.001 $\eta^2$ = 0.07 | $F$ (1, 455) = 17.0, $p$ < 0.001 $\eta^2$ = 0.03 | $F$ (1, 455) = 35.4, $p$ < 0.001 $\eta^2$ = 0.07 |

M, mean scores; SD, standard deviation; U.S., United States
